# Long-Term Safety of Rituximab in DLBCL Patients With Hepatitis B-Related Cirrhosis: A Retrospective Case Series

**DOI:** 10.3389/fmed.2022.890339

**Published:** 2022-05-30

**Authors:** Zaiwei Song, Yi Ma, Dan Jiang, Rongsheng Zhao, Fei Dong

**Affiliations:** ^1^Department of Pharmacy, Peking University Third Hospital, Beijing, China; ^2^Department of Hematology, Lymphoma Research Center, Peking University Third Hospital, Beijing, China

**Keywords:** diffuse large B cell lymphoma, cirrhosis, rituximab, hepatitis B virus, reactivation

## Abstract

**Objective:**

Chemotherapy regimens containing rituximab (RTX) have been extensively used to treat diffuse large B cell lymphoma (DLBCL). However, data looking at long-term safety of DLBCL patients with hepatitis B-related cirrhosis are still lacking. This study aims to report the safety and outcomes of RTX administration in DLBCL patients with hepatitis B-related cirrhosis.

**Methods:**

A retrospective case series was designed and implemented, using data from January 1, 2011 to December 31, 2020. Consecutive patients who were diagnosed with DLBCL and hepatitis B-related cirrhosis receiving RTX treatment were included. The primary outcomes included HBV reactivation, hepatitis flares or abnormal liver function. Survival status, the secondary outcome measure, was observed until death, loss to follow-up, or the end of follow-up, whichever occurred first.

**Results:**

A total of 8 DLBCL patients combined with hepatitis B-related cirrhosis were included in this study [4 men; median age 62.5 years (range, 44–77 years); median RTX-containing regimen course 5 (range, 2–11)]. Of them, 6 patients had current HBV infection with HBsAg-positive and anti-HBc-positive, whereas 2 patients had previously resolved HBV infection with HBsAg-negative and anti-HBc-positive. The HBV reactivation was observed in only one patient, who received 11 courses of RTX-containing immunochemotherapies within 15 months. No hepatitis flares or abnormal liver function occurred in any patients included. All patients received standardized antiviral therapy for a lifelong time. Of 8 patients included, 3 patients died, and 1 patient was lost to follow-up, and the median overall survival among patients was 39 months (range, 7–82 months).

**Conclusion:**

The findings provide support for the concept that, on the premise of standardized and valid management strategy, RTX containing regimens may be a safe option for use as the treatment of DLBCL patients combined with hepatitis B-related cirrhosis.

## Introduction

The anti-CD20 monoclonal antibody rituximab (RTX), first approved for clinical use in 1997, has changed the standard of care for patients with various non-Hodgkin lymphoma (NHL) and non-malignant immune-mediated diseases (1, 2). It has been well recognized that the addition of RTX to standard chemotherapy regimens substantially improves both response rates and survival outcomes in patients with diffuse large B cell lymphoma (DLBCL), which represents the major subtype of NHL worldwide (3, 4). Over recent years, RTX has been extensively used as a mainstay therapeutic agent for DLBCL. For example, an R-CHOP regimen comprising RTX, anthracyclines, cyclophosphamide, vincristine, and prednisone is established as the first-line treatment for DLBCL by the National Comprehensive Cancer Network (NCCN) clinical practice guideline (5).

RTX is of great clinical importance, whereas a close link has been established between RTX therapy and hepatitis B virus (HBV) reactivation (6–8). Patients with HBV reactivation may postpone scheduled chemotherapy or present with abnormal liver function, leading to adverse effects on treatment outcome for the primary disease. It is estimated that 296 million people worldwide are living with chronic HBV infection in 2019 (9). Among individuals with chronic HBV infection, up to 40% of untreated patients chronic progress to cirrhosis, which may lead to liver failure, hepatocellular carcinoma and even death (10). Globally, cirrhosis caused more than 1.32 million deaths in 2017, and hepatitis B remains one of leading causes of cirrhosis worldwide (11). Thus, cirrhosis is a major cause of morbidity and mortality burden across the world, and imposes a substantial health problem on many countries (11).

In particular, with the largest population in the world, China still faces the severe problem of HBV infection and cirrhosis. Currently, as many as 7 million (0.5%) of the total Chinese population lives with cirrhosis (12). And it is worth mentioning that, the prevalence of HBV in China (6.2%) is much higher than that in developed countries (0.71-1.17%) (12–15), so there is still plenty of room for growth in the scale of cirrhosis. Therefore, the clinical management of RTX therapy in patients with hepatitis B-related cirrhosis is still posing a major challenge.

Severe and even fatal HBV reactivation has been described in patients with previous HBV infection undergoing RTX-containing chemotherapy. Anti-CD20 monoclonal antibody therapy (e.g., RTX) has been widely recognized as a very high risk for HBV reactivation in patients with chronic infection, which means that the chance of HBV reactivation is >20% (16, 17). Additionally, previous studies have confirmed a high risk of HBV reactivation in Asian lymphoma patients with previously resolved HBV infection (18–20). Nevertheless, none of the published studies have evaluated the safety of RTX therapy and the risk of HBV reactivation in patients with hepatitis B-related cirrhosis. Currently, in real clinical practice, there still exist knowledge gaps concerning the safety of RTX therapy in lymphoma patients with hepatitis B-related cirrhosis.

Herein, the objective of this retrospective case series was to report the safety and outcomes of RTX administration in DLBCL patients with hepatitis B-related cirrhosis. We aimed to fill the gaps between knowledge and clinical practice, and provide evidence for further RTX therapy in patients with hepatitis B-related cirrhosis.

## Patients And Methods

### Study Design and Patients

A retrospective case series was designed and implemented. During a 10-year period from January 1, 2011 to December 31, 2020, the study population included consecutive patients who were diagnosed with DLBCL and hepatitis B-related cirrhosis and underwent RTX treatment at the Lymphoma Research Center of the Peking University Third Hospital. This retrospective study was approved by the hospital institutional review board (No. IRB00006761-M2022059). The requirement for informed patient consent was waived, because it was determined that this study did not directly involve human participants and was a secondary analysis of existing data of deidentified patients.

The diagnosis and classification of DLBCL were mainly based on a constellation of clinical, morphologic, immunophenotypic, and molecular genetic features. The histological classification was based on the 2016 World Health Organization classification standard of hematopoiesis and lymphoid tissue tumor diseases (21). The clinical staging was derived from the Ann Arbor staging system (22, 23). The diagnosis of hepatitis B-related cirrhosis was based on the etiology, history, clinical manifestations, complications, treatment process, laboratory tests, imaging, and histology. The Child-Pugh scoring system was used to classify cirrhosis patients into grade A, B or C. A total score of 5 to 6 was considered grade A, 7 to 9 was considered grade B, and 10 to 15 was considered grade C (24). In grade A, the cirrhosis was compensated, while in grade B or C, it was decompensated.

### Data Collection and Follow-Up

Demographic, clinical, and follow-up data were collected with database templates from electronic medical records. The demographic and basic characteristics collected included medical record number, patient name, age, sex, classification and staging of DLBCL, Child–Pugh score, and HBV serum markers (HBsAg, anti-HBs, HBeAg, anti-HBe, anti-HBc, and HBV DNA burden) at baseline at admission to the hospital for DLBCL diagnosis. The patients' treatment process was recorded, including the regimen and course of chemotherapy (immunochemotherapy), the course of radiotherapy, the dosage and course of RTX therapy, and the dosage and course of antiviral drugs.

HBV serum markers (HBV DNA burden and HBsAg) were collected before and 1 to 3 months after administrating the first dose of RTX, as well as after prolonged follow-up. In addition, liver function tests were recorded before and 1–3 months after administrating the first dose, including alanine aminotransferase (ALT), aspartate aminotransferase (AST), total bilirubin (T-BIL), albumin (ALB), prothrombin time (PT) and prothrombin activity (PTA). Survival status was ascertained from follow-up medical clinic records of the hospital information system or by contacting the patients or their families by telephone. All patients were followed up from the diagnosis of DLBCL until death, loss to follow-up, or the end of follow-up on 10 October, 2021, whichever occurred first.

### Outcome Measures

The primary outcome measures were HBV reactivation, and hepatitis flares or abnormal liver function. (1) HBV reactivation, whose definition is in line with the American Association for the Study of Liver Diseases (AASLD) 2018 hepatitis B guidance and Chinese consensus 2021 (25, 26). For HBV infection patients (HBsAg-positive, anti-HBc-positive), HBV reactivation is reasonably defined as 1 of the following: (i) a ≥100-fold increase in HBV DNA compared to the baseline level, (ii) reverse HBV DNA positivity in a patient with baseline HBV DNA negativity, or (iii) HBV DNA ≥ 10,000 IU/ml if the baseline level is not available. For previously resolved HBV infection patients (HBsAg-negative, anti-HBc-positive), the following criteria are reasonable for HBV reactivation: (i) HBV DNA is detectable, or (ii) reverse HBsAg seroconversion occurs (reappearance of HBsAg). (2) Hepatitis flares or abnormal liver function. According to the AASLD guidance (25), a hepatitis flare is reasonably defined as an ALT increase to ≥3 times the baseline level and >100 U/L. Based on the Common Terminology Criteria for Adverse Events established by the American National Cancer Institute (NCI-CTC) (27), abnormal liver function was defined as an ALT or AST increase to ≥3 times the baseline level, a T-BIL increase to ≥1.5 times the baseline level, or hypoalbuminemia with ALB <30 g/L.

The secondary outcome measure was overall survival. Overall survival was calculated based on the date of diagnosis and the date of death, the loss to follow-up, or the last follow-up for any cause (months).

### Statistical Analysis

The data collected were recorded using Microsoft Excel 2020 software. Categorical variables were commonly represented as counts or frequencies. Continuous variables were expressed as the median (range). Then, descriptive analysis was performed using Microsoft Excel 2020 software. Statistical analysis of overall survival was performed using IBM SPSS, version 26 software (IBM Corp., Armonk, N.Y., USA).

## Results

### Patient Characteristics

A total of 8 patients receiving RTX-containing chemotherapy were included in this study, of whom all were diagnosed with DLBCL combined with hepatitis B-related cirrhosis. In addition, one patient had hepatocellular carcinoma (HCC). The median age of the included patients was 62.5 years (range, 44-77 years). Among the 8 patients, 5 patients had compensatory cirrhosis with Child–Pugh grade A, whereas 3 patients had decompensated cirrhosis with Child–Pugh grade B. At admission, 6 patients had current HBV infection with HBsAg-positive and anti-HBc-positive, of whom 3 patients had serum HBV DNA levels > 1,000 IU/mL. Two patients had previously resolved HBV infection with HBsAg-negative and anti-HBc-positive. The median chemotherapy course was 7 (range, 3-12), the median RTX-containing regimen course was 5 (range, 2-11), and the single dose of RTX administration was consistently 375 mg/m^2^. The characteristics and the course of RTX treatment of the included patients are shown in Table 1.

**Table 1:** The characteristics and course of RTX treatment of the included patients.

**Patient**	**Sex**	**Age (years)**	**Diagnosis**	**C-P^**a**^**	**Baseline of HBV serum markers**						**History of chemotherapy**	**Number of RTX**
											**or radiotherapy**	**therapy courses**
					**HBsAg**	**Anti-HBs**	**HBeAg**	**Anti-HBe**	**Anti-HBc**	**HBV DNA^**b**^**		
1	M	70	DLBCL IVA	5^c^	Positive	Negative	Negative	Positive	Positive	Negative	CHOP#1, R-CHOP#1, R-BEACOP#3, R-EACOP#1, ESHAP#1	5
2	M	44	DLBCL IVA	5	Positive	Negative	Negative	Positive	Positive	3,740 IU/mL	R-CHOP#1, R-BEACOP#3, R-CHOPE#2, R-GDP#1	7
3	F	63	DLBCL	5	Positive	Negative	Positive	Negative	Positive	Negative	CHO#2, Radiotherapy#22, R-CHOP#1, R-COP#1	2
4	F	51	DLBCL IVA	5	Positive	Negative	Positive	Positive	Positive	40,000 IU/mL	CHOP#1, EPOCH#1, R-EPOCH#1, R-FC#4	5
5	F	62	DLBCL IVA	7^d^	Positive	Negative	Positive	Positive	Positive	1,640 IU/mL	CHOP#2, EPOCH#1, GDP-Lenalidomide#2, V-CHP#1, DICE#1, FC#1, ABVD#2, R-GDP#2	2
6	M	49	DLBCL IIIB	8^d^	Positive	Negative	Negative	Negative	Positive	Undetectable^e^	R-COP#5, R#1, GD#1	6
7	F	77	DLBCL IVA	5	Negative	Negative	Negative	Positive	Positive	NA^f^	R-COP#1, R-EPOCH#5, R#1, R-MTX#1, R-EPOCH#1, R-COP#1, R-CHOP#1	11
8	M	70	DLBCL IIIA	7^d^	Negative	Negative	Negative	Positive	Positive	Negative	R-CHOP#3	3

*^a^Child–Pugh score. ^b^The cutoff values of HBV DNA positivity were 1,000 (from Jan. 2010 to Dec. 2013), 100 (from Jan. 2014 to Apr. 2016), and 30 IU/ml (from May 2016 to the end of the follow-up). ^c^Combined with hepatocellular carcinoma (HCC). ^d^Decompensated cirrhosis. ^e^Below the lower limit of detection of serum HBV DNA burden. ^f^NA, not available*.

### HBV Reactivation During Follow-Up

For three patients with baseline positive HBV DNA at admission, antiviral therapy, including entecavir (ETV) in combination with or without adefovir dipivoxil (ADV), was administered until HBV DNA turned negative before the initiation of chemotherapy. For all patients, the serum levels of HBsAg and/or HBV DNA were confirmed negative before administrating RTX. For six infection patients with HBsAg-positive and anti-HBc-positive, HBV DNA remained negative or undetectable, and HBV reactivation was not observed. For two previously resolved infection patients with HBsAg-negative and anti-HBc-positive, one patient remained HBsAg-negative and HBV DNA-negative, whereas HBV reactivation was observed in the other patient. For the latter, the patient received 11 courses of RTX-containing immunochemotherapies within 15 months, whose courses of RTX therapy were the most. In addition, the patient's reappearance of HBsAg and reverse HBV DNA positivity might be associated with the progression of the primary disease, since the reversal of HBV DNA and HBsAg occurred after lymphoma progression [with a lactate dehydrogenase (LDH) level of 770 U/L]. The serum HBV DNA and HBsAg of the included patients before and after RTX administration are shown in Table 2.

**Table 2:** The serum levels of hbv dna and hbsag in the included patients before and after rtx administration.

**Patient**	**HBV DNA** ^**c**^			**HBsAg**		**HBV reactivation**
	**Before RTX**	**1–3 month post-RTX**	**Long-time post-RTX**	**Before RTX**	**1–3 month post-RTX**	
1^*a*^	Negative	Negative	Negative	Positive	Positive	No
2	Negative	Negative	Negative	Positive	Positive	No
3	Negative	Negative	Undetectable^e^	Positive	Positive	No
4	Negative	Negative	Negative	Positive	Positive	No
5^b^	Undetectable	Undetectable	Undetectable	Positive	Positive	No
6^b^	Undetectable	Undetectable	Undetectable	Positive	Positive	No
7	NA^d^	NA	44,4000 IU/mL	Negative	Negative	Yes
8^b^	Negative	Negative	Negative	Negative	Negative	No

*^a^Combined with hepatocellular carcinoma (HCC). ^b^Decompensated cirrhosis. ^c^The cutoff values of HBV DNA positivity were 1,000 (from Jan. 2010 to Dec. 2013), 100 (from Jan. 2014 to Apr. 2016), and 30 IU/mL (from May 2016 to the end of the follow-up). ^d^NA: Not available. ^e^Below the lower limit of detection of serum HBV DNA burden*.

### Hepatitis Flares or Abnormal Liver Function During Follow-Up

**Figure 1:**
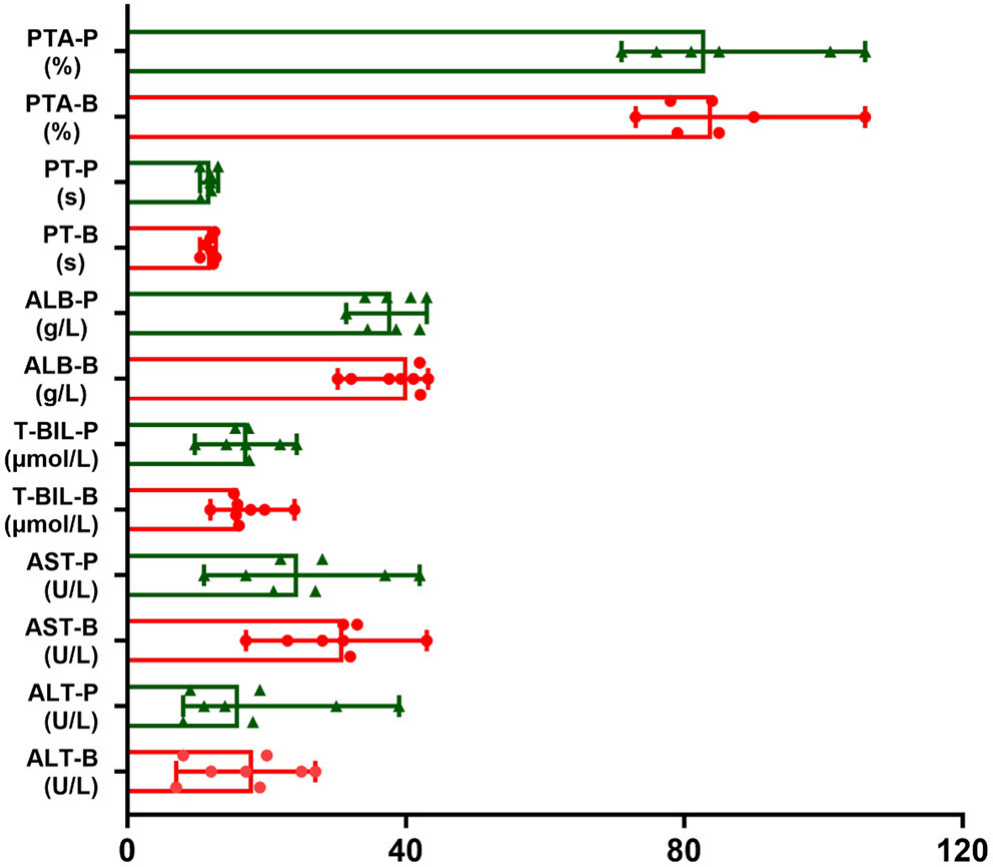
The trends of liver function tests of included patients before and after RTX administration. The red one is value of liver function before RTX treatment (B). The green one is value of liver function post RTX treatment for 1–3 months (P). Data were expressed as median and minimum and maximum values.

No hepatitis flares or abnormal liver function occurred in any patients. Regarding the serum levels of ALT, AST and T-BIL, no patient developed elevated ALT, AST, and T-BIL. Regarding the serum level of ALB, two patients' ALB levels were reduced from 41.1 g/L and 39.3 g/L to 34.1 g/L and 31.4 g/L, respectively, but none of them developed hypoalbuminemia. Regarding PT and PTA, compared with the baseline level before administrating RTX, no obvious change was observed. The liver function tests of the included patients before and after RTX administration are shown in Table 3 and Figure 1.

**Table 3:** Liver function tests of included patients before and after RTX administration.

**Patient**	**ALT (U/L)**		**AST (U/L)**		**T-BIL (μmol/L)**		**ALB (g/L)**		**PT (s)**		**PTA (%)**	
	**Before**	**1–3 month**	**Before**	**1–3 month**	**Before**	**1–3 month**	**Before**	**1–3 month**	**Before**	**1–3 month**	**Before**	**13 month**
	**RTX**	**post-RTX**	**RTX**	**post-RTX**	**RTX**	**post-RTX**	**RTX**	**post-RTX**	**RTX**	**post-RTX**	**RTX**	**post-RTX**
1^a^	12	11	17	17	15.6	17.5	37.6	38.6	12.7	13	73	71
2	27	39	33	42	11.9	9.7	42	43	NA^c^	10.5	NA	101
3	20	18	31	27	15.8	15.5	42.1	40.7	10.4	10.4	106	106
4	25	30	32	37	24	24.3	43.2	42	12.3	12	78	76
5^b^	17	14	23	22	15.3	21.9	41.1	34.1	12	NA	84	NA
6^b^	8	8	31	11	16	17	39.3	31.4	12.5	NA	79	NA
7	7	9	28	21	17.7	17.4	32.1	34.5	11.9	11.9	85	85
8^b^	19	19	43	28	19.7	14.2	30.2	37.3	11.2	11.9	90	81

*^a^ombined with hepatocellular carcinoma (HCC). ^b^Decompensated cirrhosis. ^c^NA, not available*.

### Antiviral Prophylaxis and Clinical Outcomes

In line with clinical practice guidelines from the American Society of Clinical Oncology (ASCO) (28) and AASLD (25), antiviral prophylaxis is recommended for chronic infection patients with positive HBsAg or resolved infection patients receiving anti-CD20 antibody therapy. All included patients were at high risk of HBV reactivation and received standardized antiviral therapy for a lifelong time. Among them, ETV prophylaxis was used for 6 patients, ADV prophylaxis was used for one patient, and ETV and ADV were combined for one patient. During follow-up, 4 (50%) patients survived, 1 (12.5%) patient was lost to follow-up, and 3 (37.5%) patients died. HBV reactivation occurred after tumor progression in one of the deceased patients. The median overall survival (mOS) of the included patients was 39 months (range, 7–82 months). The antiviral prophylaxis and clinical outcomes of the included patients are shown in Table 4.

**Table 4:** Antiviral prophylaxis and clinical outcomes of the included patients.

**Patient**	**Antiviral prophylaxis**	**Clinical outcomes**	**Overall survival**	**Description of cause of death**
1^a^	ADV^c^ 10 mg/day	Died	10 months	The patient died of lymphoma recurrence, hepatic failure, hepatic encephalopathy and gastrointestinal bleeding, whereas the HBV DNA still remained negative.
2	ETV^d^ 0.5 mg/day	Alive	80 months	
3	ETV 0.5 mg/day	Alive	68 months	
4	ETV 0.5 mg/day ADV 10 mg/day	Loss to follow-up	13 months	The patient was diagnosed as DLBCL in Nov. 2015, and the loss of follow-up occurred in Dec. 2016.
5^b^	ETV 0.5 mg/day	Alive	60 months	
6^b^	ETV 0.5 mg/day	Died	7 months	The patient died of lymphoma progression, whereas the HBV DNA still remained negative.
7	ETV 0.5 mg/day	Died	18 months	The HBV reactivation occurred after the tumor progression in the patient, and finally he died of lymphoma progression.
8^b^	ETV 0.5 mg/day	Alive	82 months	

*^a^Combined with hepatocellular carcinoma (HCC). ^b^Decompensated cirrhosis. ^c^ADV, adefovir dipivoxil; ^d^ETV, entecavir*.

## Discussion

### Strength and General Findings of This Study

To our knowledge, this is the first study to report the long-term safety of RTX in patients with hepatitis B-related cirrhosis. HBV infection has been shown to increase the risk of developing DLBCL and other NHL, and the incidence of HBV infection is higher in DLBCL patients than in other malignancy patients (29–31). Meanwhile, the size of the population with hepatitis B-related cirrhosis is not to be underestimated. But there is extremely limited information about long-term outcomes of rituximab administration in patients with hepatitis B-related cirrhosis. Therefore, we paid more attention to DLBCL patients with hepatitis B-related cirrhosis in this present study. This study is unique in its included hepatitis B-cirrhosis population with both current HBV infection and previously resolved HBV infection, whereas most other publications have focused on lymphoma patients with previously resolved HBV infection and without cirrhosis (18–20).

Our findings pinpointed to a few salient points among the case series. First, the close link has not been established between DLBCL patients with hepatitis B cirrhosis and HBV reactivation or hepatitis flares, which is in agreement with a former study in patients with hepatitis C-related cirrhosis (32). Besides, the only case of HBV reactivation suggests that long-term and frequent RTX therapy may contribute to increasing the risk of HBV reactivation, and consequently, more effective prevention and close monitoring are warranted. Third, this study provides support for the concept that clinical management strategies, including screening prior to treatment and early and lifelong antiviral treatment, contribute to avoiding HBV-related liver complications and thereby improving patients' long-term outcomes.

### Recommendation for Clinical Practice

From a clinician or clinical pharmacist's point of view, concerns about the risk of HBV reactivation always limit RTX use in hepatitis B-related cirrhosis, leading to adverse effects on patients' outcome. Therefore, the clinical treatment of DLBCL patients with hepatitis B-related cirrhosis still remains a challenge we have to face. Herein, we tried to discuss recommendations regarding the clinical management of HBV reactivation in DLBCL patients with hepatitis B-related cirrhosis, who usually need to receive first-line RTX-containing chemotherapy.

First, we strongly recommend that the prevention of HBV reactivation begin with patient screening before the initiation of therapy (33). The baseline HBV serum markers (e.g., HBV DNA burden, HBsAg) should be tested and recorded. Second, after screening, we think it is necessary to initiate antiviral prophylaxis before RTX-containing therapies are given (33). Especially for chronic infection patients who are HBsAg-positive and HBV DNA-positive, antiviral prophylaxis should be initiated until HBV DNA is undetectable or negative. Third, it is well recognized that nucleoside analogs (NAs) have been shown to decrease the risk of HBV reactivation. In line with relevant clinical guidelines or research evidence (5, 25, 34), we would like to recommend ETV or tenofovir (especially ETV) as the standard agent for antiviral prophylaxis.

Furthermore, although most guidelines recommend that the antiviral duration is at least 12 months after the end of immunochemotherapy (25, 28, 35), we would like to recommend life-long antiviral prophylaxis for DLBCL patients with hepatitis B-related cirrhosis. The reason for extending the duration of antiviral prophylaxis is long-term immunosuppression of hematological malignancy patients, and HBV reactivation has been reported at 55 months after the completion of chemotherapy (36).

Last but not least, regarding the monitoring and follow-up, the monitoring of liver function tests and HBV serum markers is recommended every 1-3 months after the first year of chemotherapy completion, followed by every 3-6 months. It is worth mentioning that ALT elevations may occur earlier than the increase in HBV DNA replication (37), and monitoring of ALT levels may contribute to suggestive HBV reactivation. Thus, we recommend monitoring liver enzymes and HBV serum markers at the same time.

## Limitations

Our findings must be interpreted with caution considering several limitations. First, this study was limited by a small sample size, although we included consecutive patients during the 10-year duration period. Second, although our single-center experience suggested that liver injury usually occurred within 1-3 months after RTX-containing chemotherapy (38), the possibility of delayed liver injury could not be completely excepted. In addition, we only included Chinese patients with hepatitis B-related cirrhosis, while the genotypes of HBV may vary from one geographic area to another, and the pathogenic differences between HBV genotypes should be taken into consideration (39). Future larger prospective validation studies, including different patient populations, are still warranted to draw definitive conclusions.

## Conclusions

To conclude, the findings from this retrospective case series provide support for the concept that RTX containing regimens may be a safe option for use as the treatment of DLBCL patients combined with hepatitis B-related cirrhosis. Clinical management strategies, including screening for HBV serum markers prior to commencing treatment, life-long antiviral prophylaxis and careful and regular monitoring, are of the utmost importance to avoid HBV reactivation.

## Data Availability Statement

The original contributions presented in the study are included in the article/supplementary material, further inquiries can be directed to the corresponding author.

## Ethics Statement

The studies involving human participants were reviewed and approved by Peking University Third Hospital Institutional Review Board.

## Author Contributions

Conceptualization: ZS and FD. Data curation: ZS, YM, and FD. Formal analysis, investigation, and methodology: ZS and YM. Funding, acquisition, project administration, and validation: FD. Visualization: YM and DJ. Writing—original draft: ZS. Supervision and writing—review and editing: RZ and FD. All authors contributed to the article and approved the submitted version.

## Funding

This work was supported by the Beijing Medical Award Foundation.

## Conflict of Interest

The authors declare that the research was conducted in the absence of any commercial or financial relationships that could be construed as a potential conflict of interest.

## Publisher's Note

All claims expressed in this article are solely those of the authors and do not necessarily represent those of their affiliated organizations, or those of the publisher, the editors and the reviewers. Any product that may be evaluated in this article, or claim that may be made by its manufacturer, is not guaranteed or endorsed by the publisher.
